# IL-18-primed NK cells recruit dendritic cells and potentiate tumor therapy mediated by PD-1 blockade

**DOI:** 10.3389/fonc.2025.1533808

**Published:** 2025-03-17

**Authors:** Yoshiya Ohno, Haruki Okamura, Hideo Yagita, Toshiyuki Tanaka

**Affiliations:** ^1^ Laboratory of Immunobiology, School of Pharmacy, Hyogo Medical University, Kobe, Hyogo, Japan; ^2^ Department of Psychoimmunology, School of Medicine, Hyogo Medical University, Nishinomiya, Hyogo, Japan; ^3^ Department of Immunology, Juntendo University School of Medicine, Tokyo, Japan

**Keywords:** IL-18, immune checkpoint inhibitor, PD-1, NK cells, cDC1

## Abstract

The success of cancer immunotherapy depends on the effective coordination of innate and adaptive immunity. We previously reported that IL-18 potentiates the therapeutic effects of immune checkpoint inhibitors in mouse models. Here, we report that IL-18-primed natural killer (NK) cells enhanced the antitumor effects of anti-PD-1 antibodies by mobilizing type 1 conventional dendritic cells (cDC1s) to tumor sites and promoting type 1 immune responses. IL-18-primed NK cells had a premature phenotype, and expressed chemokines involved in cDC1 mobilization. In a combination treatment with IL-18 and anti-PD-1 antibody, NK cell depletion inhibited cDC1 mobilization and abrogated the therapeutic effects. Additionally, adoptive transfer of IL-18-primed NK cells induced cDC1 mobilization and enhanced the therapeutic effects of anti-PD-1 antibodies. IL-18 also increased IL-12 mRNA expression in DCs and IL-12 blood levels, and IL-12 upregulated XCL1 expression in NK cells. These results suggest that IL-18 primes NK cells and enhances the therapeutic effects of immune checkpoint inhibitors by promoting a feed-forward loop involving DCs.

## 1 Introduction

Through modulation of regulatory pathways in T cells, immune checkpoint blockade (ICB) therapy enhances antitumor immune responses and has achieved unprecedented improvements in tumor control in various types of human cancers (1). However, only a subset of patients respond to these therapies, and a substantial fraction of cancers do not respond to immune checkpoint inhibitors (1). The success of ICB therapy depends to a large degree on the immunological state of the tumor microenvironment (2, 3). Inflammatory infiltrates in the tumor microenvironment determine the responsiveness to ICB therapy, and tumor-infiltrating natural killer (NK) cells (4–6) accelerate antitumor immunity through their interaction with dendritic cells (DCs) (7, 8) to initiate the cancer-NK cell immunity cycle (9). These studies have highlighted the roles of NK cells in modulating cDC1 activity within the tumor microenvironment. NK cells produce chemoattractants, such as CCL5 and XCL1, that recruit cDC1s to tumor sites (7), meanwhile, tumor-derived factors, such as PGE2, can suppress NK cell viability and chemokine production. NK cells also contribute to cDC1 abundance through FLT3L production, enhancing DC-mediated antitumor immunity (8). However, the exact molecular pathways driving NK-DC interactions and their role in the cancer-NK cell immunity cycle to promote tumor-destructive type 1 immune responses remain to be fully elucidated.

We previously showed that IL-18 strongly augments the potency of ICB therapy in preclinical murine models through NK cell-dependent mechanisms (10). IL-18, a member of the IL-1 family of cytokines that enhances IFN-γ production from activated T and NK cells, has been shown to induce type 1 immune responses in concert with IL-12 (11, 12). Previous studies have shown that IL-18 has unique NK cell-priming properties (13). In concert with other cytokines such as IL-12 and IL-15, IL-18 induces memory-like NK cells with enhanced cytokine production ability that can activate DCs (14–17). Treatment of tumor-bearing mice with a combination of anti-CTLA-4 mAb and anti-PD-L1 mAb together with IL-18 led to the accumulation of activated NK cells, and depletion of either NK cells or CD8^+^ T cells abrogated the therapeutic effects of ICB plus IL-18 (10), indicating essential roles of these lymphocyte populations. While critical roles of CD8^+^ T cells in ICB therapy are well-established, mechanisms underlying how activated NK cells mediate enhanced therapeutic effects observed with ICB plus IL-18 remain elusive.

In this study, we focused on IL-18-primed NK cells and set out to elucidate how they enhance the anti-tumor effects of ICB therapy. We investigated the properties of IL-18-primed NK and their role in promoting antitumor effector cell mobilization in mouse models.

## 2 Materials and methods

### 2.1 Mice

BALB/c and C57BL/6 mice were purchased from SLC Japan (Hamamatsu, Japan). Mice were housed in specific pathogen-free conditions and used at 6–14 weeks old. All animal experiments were approved by the institutional animal care and use committee at Hyogo University of Health Sciences (approval numbers 2008-03, 2010-09, 2013-02, and 2013-22).

### 2.2 Cell culture

CT26 colorectal cancer cells were purchased from ATCC. B16D8 mouse melanoma cells were obtained from Dr. Yamada (Department of Pathology, Hyogo Medical University). All cells were cultured in RPMI 1640 medium (Sigma) supplemented with 2 mM L-glutamine (Gibco), 100 units/mL penicillin, 100 µg/mL streptomycin (Gibco), 10 mM HEPES (Gibco), 1 mM sodium pyruvate (Gibco), minimum essential medium/nonessential amino acids (MEM/NEAA) (Gibco), and 10% heat-inactivated FBS (Gibco) in humidified CO_2_ incubators at 37°C. All cells were used within four weeks of culture and were tested for mycoplasma contamination using a DAPI (Sigma) staining method.

### 2.3 Generation of β2-microglobulin-deficient cells

Beta 2-microglobulin-deficient CT26 (CT26-Δβ2m) cells were generated with CRISPR/Cas9 technology. The synthetic oligos for β2m-targeting guide RNA (forward: 5’-CACCGAGTATACTCACGCCACCCAC-3’; reverse: 5’-AAACGTGGGTGGCGTGAGTATACTC-3’) were hybridized, and the dsDNA was cloned into pX458 plasmid (18) (Addgene #48138) to obtain β2m-pX458 plasmid, which simultaneously expresses GFP, β2m-gRNA, and Cas9. The β2m-pX458 was transfected into CT26 cells with transfecting reagent (TaKaRa) according to the manufacturer’s instructions. GFP-positive β2m-pX458-transfected CT26 cells were sorted and collected as a single-cell suspension by FACS AriaIIIu cell sorter (Becton Dickinson). Deletion of the β2m gene was confirmed by flow cytometry. CT26-Δβ2m clones lacked β2m expression following stimulation with IFN-γ (10 μg/mL, 24 h).

### 2.4 Tumor models

BALB/c mice were injected intraperitoneally with CT26 cells (5×10^4^ cells). The survival of mice harboring peritoneal dissemination of CT26 cells was monitored daily. C57BL/6 mice were injected in the flank subcutaneously with B16D8 cells (1×10^5^ cells). Tumor growth was measured every 2–3 d with a caliper. Tumor mass was calculated by the formula: tumor mass (mm^3^) = length (mm) × width (mm)^2^ × 1/2. Tumor-bearing mice were administered intraperitoneally with a control antibody or anti-PD-1 mAb (RMP1-14, 200 μg/mouse; prepared in our laboratory) either alone or in combination with IL-18 (2 μg/mouse; GlaxoSmithKline PLC) on days 3, 7, 11, and 15 after transplantation of tumor cells (day 0). Mice were euthanized when their body weight was reduced by more than 20% compared to that of untreated mice or increased by more than 20% due to ascites in the CT26 i.p. inoculation model or tumors reached 2,000 mm^3^ or upon ulceration in s.c. implantation models. For depletion of NK cells, mice were treated with i.p. injections of either anti-CD122 mAb (TM-ß1, 500 μg/mouse; prepared in our laboratory), anti-asialo GM1 polyclonal antibody (50 μL/mouse, Wako Pure Chemicals), or anti-NK1.1 mAb (PK136, 500 μg/mouse; prepared in our laboratory). Anti-CD122 mAb and anti-asialo GM1 antibody were used in Balb/c mice, while anti-NK1.1 mAb, due to its allotype specificity, was applied exclusively to C57BL/6 mice. For neutralization of mouse IL-12, mice were treated with i.p. injections of anti-mouse IL-12p75 mAb (R2-9A5; BioXcell). In some experiments, mice were treated i.p. with FTY720 (1 mg/kg, Sigma) on days 2, 6, 10, and 14 after transplantation of tumor cells (day 0) to inhibit lymphocyte egress from secondary lymphoid tissues.

### 2.5 Isolation of PECs and TILs

PECs were recovered from the peritoneal cavity of mice i.p. inoculated with CT26 cells by washing with 5 mL of ice-cold RPMI 1640 medium containing 5% FCS. For isolation of TILs from s.c. injected B16D8 tumors, tumor tissues were mechanically minced with scissors before digestion with Liberase DH (0.26 U/mL; Roche) for 2 h at 37°C. After digestion and neutralization, the samples were passed through 70-µm nylon filters, and mononuclear cells were isolated by centrifugation through a Percoll gradient (40% and 80%). The PECs and TILs were washed once with RPMI 1640 medium containing 10% FCS and subjected to subsequent analysis. Total cell number and viability were manually quantified using a hemocytometer with trypan blue.

### 2.6 Flow cytometry

Cells were resuspended in PBS with 2% FCS and 1 mM EDTA and stained with fluorescently conjugated antibodies for 1 h on ice. The following fluorescently conjugated antibodies were purchased from BioLegend and used: anti-CD3 (145-2C11), anti-CD4 (RM4-5), anti-CD8α (53-6.7), anti-CD11b (M1/70), anti-CD11c (N418), anti-CD45 (104), anti-CD45RO (B220: RA3-6B2), anti-CD49b (Dx5), anti-NKG2D (C7), anti-DNAM-1 (10E5), anti-KLRG1 (2F1/KLRG1), anti-Ly49A (YE1/48.10.6), anti-CD103 (2E7), anti-CD206 (C068C2), anti-NK1.1 (PK136), anti-XCR1 (ZET), anti-F4/80 (BM8), anti-I-A/I-E (M5/114.15.2), and anti-IL-18Rα (A17071D). After washing the cells with PBS containing 0.2% BSA, the fluorescence intensity of individual cells was determined using a FACSAriaIIIu flow cytometer. Dead cells were stained with DAPI and excluded from the analysis. For intracellular staining, cells were reactivated with PMA (50 ng/mL; Sigma) and ionomycin (1 μg/mL; Sigma) in the presence of brefeldin A (50 ng/mL; BioLegend) for 4 h. Then, cells were fixed and permeabilized using a fixation buffer (BioLegend) and intracellular staining permeabilization wash buffer (BioLegend). The fixed and permeabilized cells were stained for 20 min at room temperature with fluorescently conjugated antibodies. Fluorescently conjugated anti-IFN-γ (XMG1.2) and anti-granzyme B (ICFC) antibodies were used (both BioLegend). After washing the cells with PBS containing 0.2% BSA, the fluorescence intensity was analyzed using a FACSAriaIIIu flow cytometer. FACSDiva software (Becton Dickinson) and FlowJo software, RRID: SCR_008520 (Becton Dickinson) were used for data acquisition and analysis, respectively.

### 2.7 Cell sorting

Cells in PECs of CT26-bearing mice treated with or without anti-PD-1/IL-18 were resuspended in PBS with 2% FCS and 1 mM EDTA and stained with fluorescently conjugated antibodies for 1 h on ice. After washing and resuspending with PBS containing 2% FCS and 1 mM EDTA, T cells (CD45^+^CD3^+^), CD8^+^ T cells (CD45^+^CD3^+^CD8^+^), B cells (CD45^+^CD3^-^B220^+^), activated NK cells (CD45^+^CD3^-^Dx5^+^B220^+^), and cDC1 (CD45^+^CD11c^+^CD103^+^XCR1^+^) were sorted by FACSAriaIIIu.

### 2.8 RT-PCR

The total RNA was isolated from cells using a NucleoSpin RNA Plus (Macherey-Nagel), and cDNA was obtained using the High Capacity cDNA Reverse Transcription Kit (Applied Biosystems). Semi-quantitative RT-PCR was performed using ExTaq Hot start version (TaKaRa) on GeneAmp PCR System 9700 (Applied Biosystems). cDNA was amplified under the following conditions for all primer sets: one cycle at 94°C for 3 min followed by 35 cycles at 94°C for 1 min, 60°C for 1 min, and 72°C for 1 min, and one cycle at 72°C for 7 min. Real-time quantitative RT-PCR was performed using the Power SYBR Green PCR Master Mix (Applied Biosystems) on a PRISM 7700 (Applied Biosystems). The expression of each mRNA was normalized to that of GAPDH mRNA. The primer sequences were as follows: *Gapdh*, 5’-GACCCCTTCATTGACCTCAAC-3’ and 5’-CTTCTCCATGGTGGTGAAGA-3’; *Ccl4*, 5’-GTCACAGTGTTTGCAGAGCAC-3’ and 5’-TGGAGAGGTACCTAGACTACG-3’; *Ccl5*, 5’-CCAGCAGCAAGTGCTCCAATCTTG-3’ and 5’-GCTGGCTAGGACTAGAGCAAGCAA-3’; *Cxcl9*, 5’-GAACGGAGATCAAACCTGCCT-3’ and 5’-TGTAGTCTTCCTTGAACGACGA-3’; *Cxcl10*, 5’-CGATGACGGGCCAGTGAGAATG-3’ and 5’-TCAACACGTGGGCAGGATAGGCT-3’; *Cxcl11*, 5’-AATTTACCCGAGTAACGGCTG-3’ and 5’-ATTATGAGGCGAGCTTGCTTG-3’; *Xcl1*, 5’-CTTTCCTGGGAGTCTGCTGC-3’ and 5’-CAGCCGCTGGGTTTGTAAGT-3’; *Ccr4*, 5’-GTCACAGTGTTTGCAGAGCAC-3’ and 5’-TGGAGAGGTACCTAGACTACG-3’; *Ccr7*, 5’-TTGCCGTGGTGGTAGTCTTC-3’ and 5’-TTCGCAGCTGCTATTGGTGA-3’; *Cxcr3*, 5’-AACCTTCCTGCCAGCCCTCT-3’ and 5’-CGAAAACCCACTGGACAGCA-3’; *Infg*, GAGGAACTGGCAAAAGGATGG-3’ and 5’-ACCTGTGGGTTGTTGACCTC-3’; and *Il12*, 5’-TGGGAGTACCCTGACTCCTG-3’ and 5’-AGGAACGCACCTTTCTGGTT-3’.

### 2.9 ELISA for serum IL-12

Serum IL-12p40 levels were determined using ELISA MAX™ Standard Set Mouse IL-12/IL-23 (p40) kit (BioLegend) according to the manufacturer’s protocol. Briefly, ELISA 96-well plates (Sumitomo Bakelite, Co.) were coated with the capture anti-mouse IL-12 antibody at 2 μg/mL in 0.05 M carbonate buffer at 4°C. After blocking with PBS containing 1% BSA, diluted serum samples and standards were added to the wells and incubated for 2 h. After washing five times with PBS containing 0.05% Tween-20, the specific binding protein was detected with a biotinylated anti-mouse IL-12 antibody (10 μg/mL) for 1 h, followed by streptavidin-HRP at 1:1000 for 1 h and then developed with the TMB High Sensitivity Substrate Solution (BioLegend). After terminating the reaction with 0.5 N H_2_SO_4_, absorbance at 450 nm was measured using a microplate reader (Molecular Devices).

### 2.10 Adoptive transfer of NK cells

NK cells were isolated on day 6 from splenocytes of CT26-bearing mice i.p. treated with or without anti-PD-1/IL-18 on day 3 using the MojoSort Mouse NK Cell Isolation Kit (BioLegend) according to the manufacturer’s instructions. Briefly, non-NK cells were labeled with the biotin-conjugated antibody cocktail, followed by incubation with magnetic streptavidin nanobeads. The untouched NK cells were collected by using a magnetic separator. The sorted NK cells (1.0×10^6^ cells) were resuspended in PBS, then adoptively i.p. transferred to CT26-bearing mice on day 3. On day 8, cDC1 accumulation was analyzed by flow cytometry. For combination therapy with anti-PD-1 and naïve or IL-18-primed NK cells, NK cells were isolated from splenocytes of naïve or IL-18-treated mice for three consecutive days. Isolated unprimed or IL-18-primed NK cells (1.0×10^6^ cells) were combined with anti-PD-1 mAb and adoptively i.p. transferred to CT26-bearing mice on days 3, 7, 11, and 15. The survival of CT26-bearing mice was monitored.

### 2.11 Human data analysis

Transcriptomic and clinical data of patients with SKCM and BRCA in The Cancer Genome Atlas (TCGA) research network database (PanCancer Atlas) were analyzed using the cBioPortal for Cancer Genomics (https://www.cbioportal.org/). The clinical outcome datasets of PD-1/PD-L1 blockade in patients with SKCM were assessed using Cancer-Immu (http://129.59.197.30:3838/Cancer-Immu/) (19). The pan-cancer analysis module was used to assess six patient cohorts (20–25). From the aggregated datasets, samples with unknown IL-18 expression status or unknown progression-free survival (PFS) status were excluded. Comparisons of IL-18 levels in responders and non-responders and Kaplan-Meier analysis in patients with high or low IL-18 expression were performed.

### 2.12 Statistical analysis

Values are expressed as mean ± SD. Two-tailed unpaired Student’s *t*-test was used to compare the two groups. Groups of three or more were compared by one-way or two-way ANOVA with Tukey’s multiple-comparisons test (for unpaired data). GraphPad Prism version 6.0 software, RRID: SCR_002798 (GraphPad) and R v4.2.1 were used to perform analyses. Statistically significant differences are indicated as follows: *, P < 0.05; **, P < 0.01; ***, P < 0.001; ****, P < 0.0001.

## 3 Results and discussion

### 3.1 NK cells are essential for IL-18 to potentiate the antitumor effects of anti-PD-1 antibodies

**Figure 1:**
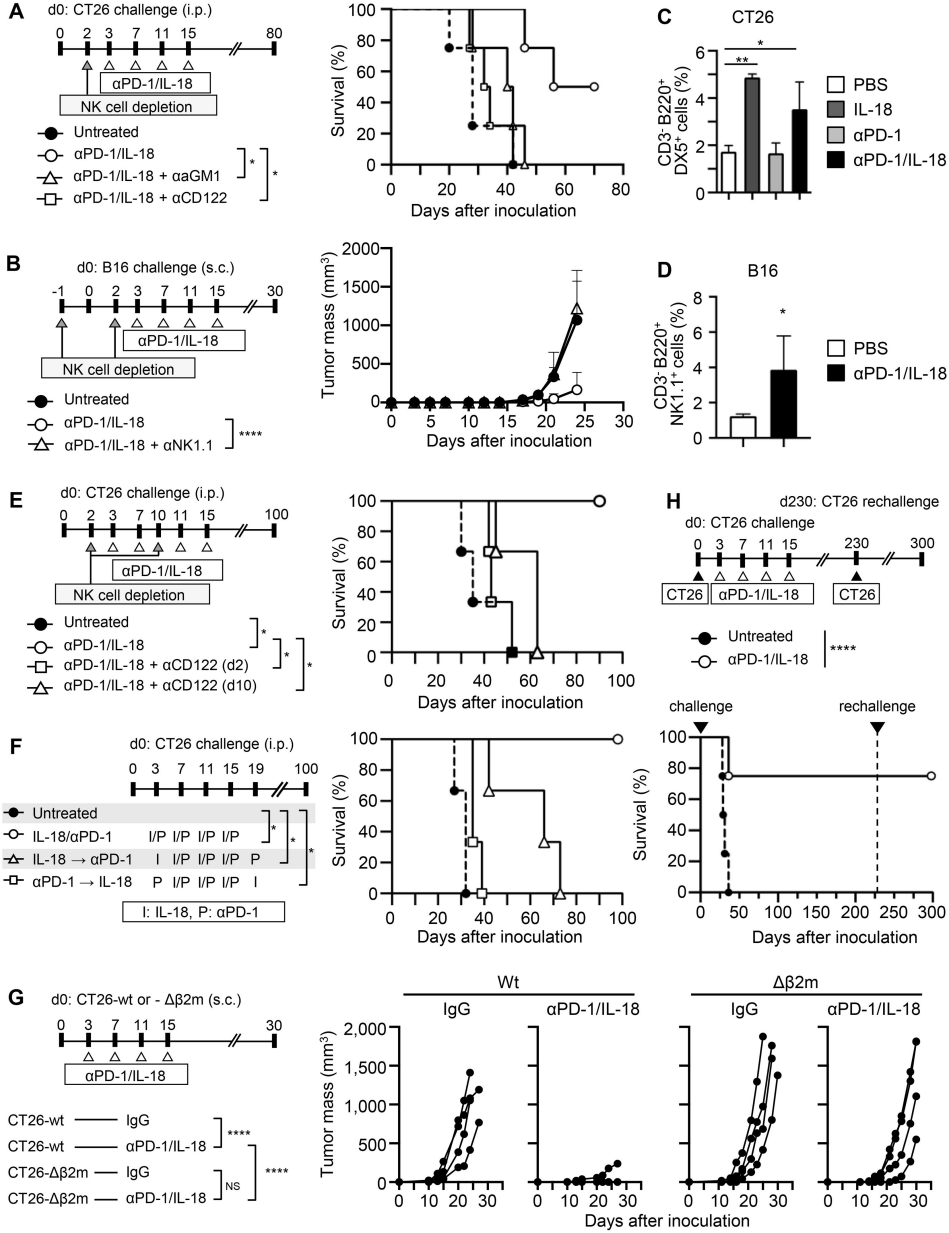
NK cells are necessary for cancer immunotherapy with anti-PD-1 mAb and IL-18. **(A, B)** Effects of NK cell depletion on the therapeutic efficacy of anti-PD-1 mAb (anti-PD-1) and IL-18. **(A)** BALB/c mice were i.p. inoculated with CT26 cells (day 0) with or without anti-PD-1 + IL-18 i.p. administration on days 3, 7, 11, and 15. NK cells were depleted by i.p. injecting either anti-asialo GM1 Ab (αaGM1) or anti-CD122 mAb (αCD122) on day 2. **(B)** C57BL/6 mice were s.c. inoculated with B16 cells with or without anti-PD-1 + IL-18 i.p. administration as in **(A)**. NK cells were depleted by i.p. injecting anti-NK1.1 mAb (αNK1.1) on days -1 and 2. **(C, D)** Effects of IL-18 on NK cell activation in the spleen of tumor-bearing mice. Activated NK cells in the spleen of CT26- **(C)** or B16- **(D)** bearing mice with or without anti-PD-1 and IL-18 i.p. administration were assessed by flow cytometry. **(E)** Effects of NK cell depletion at day 2 or day 10 of anti-PD-1 + IL-18 therapy in the CT26 tumor model. **(F)** Comparison of the efficacy of simultaneous and sequential administration of anti-PD-1 and IL-18 in the CT26 tumor model. **(G)** Comparison of the therapeutic efficacy of anti-PD-1 + IL-18 in the wild-type (wt) and β2-microglobulin-deficient (Δβ2M) CT26 tumor models. BALB/c mice were s.c. inoculated with CT26-wt or -Δβ2M (day 0) with or without anti-PD-1 + IL-18 i.p. administration on days 3, 7, 11, and 15. **(H)** BALB/c mice were i.p. inoculated with CT26 and treated with anti-PD-1 + IL-18 as in **(A)**. Mice cured with anti-PD-1 + IL-18 i.p. treatments (N=4) were re-challenged with CT26 on day 230, and their survival was observed. Data were analyzed using the log-rank test **(A, E, F, H)**, ANOVA with multiple comparisons (Tukey *post hoc* analysis) **(B, C, G)** and unpaired Student’s t-test **(D)**. Error bars indicate mean ± SD. ****, P < 0.0001; **, P < 0.01; *, P < 0.05.

We previously reported that IL-18 potentiates the therapeutic effects of ICB (anti-CTLA-4 or anti-PD-L1) on colorectal cancer CT26 and melanoma B16 metastasis *in vivo*, and B220^+^ NK cells play an essential role in this action of IL-18 (10). Here, we observed that IL-18 also enhanced the antitumor effect of anti-PD-1 (**Supplementary Figure S1A**) and analyzed the role of NK cells in the enhancement of the therapeutic effects of anti-PD-1 by IL-18. Depletion of NK cells after tumor inoculation by specific antibodies against either asialoGM1, CD122, or NK 1.1 before the start of the therapy nearly abolished the therapeutic effects of the combination of anti-PD-1 with IL-18 (anti-PD-1/IL-18) against CT26 (**Figure 1A**) and B16 (**Figure 1B**). Analysis of NK cells in the spleen revealed a significant increase in the percentage of activated B220^+^ NK cells in CT26-bearing mice treated with IL-18 alone or in combination with anti-PD-1 (**Figure 1C**). The combination treatment did not result in a further increase compared to IL-18 alone, suggesting that the therapeutic effects of IL-18 and anti-PD-1 are mediated not only by the enhancement of B220^+^ NK cell accumulation but also by additional mechanisms. The increased percentage of activated B220^+^ NK cells was not observed in CT26-bearing mice treated with anti-PD-1 alone. In B16-bearing mice, a similar increase in activated NK cells in the spleen was observed when these mice were treated with anti-PD-1/IL-18 (**Figure 1D**). Depletion of NK cells on day 10 after tumor inoculation also abolished the potentiating effects of IL-18 on anti-PD-1 treatment (**Figure 1E**). The expression of checkpoint molecules (PD-1, Tim3, TIGIT, and LAG3) in NK cells in peritoneal exudate cells (PECs) in the spleen and lymph nodes of CT26-bearing mice on day 6 following tumor inoculation was barely detectable or very low. However, PD-1 and TIGIT expression were detected in NK cells and CD8 T cells at the tumor site on day 13 in these mice (**Supplementary Figures S1B–D**).

Next, we administered IL-18 and anti-PD-1 at different time points and examined their therapeutic effects in the CT26 model. The results showed that when IL-18 was administered before anti-PD-1, the antitumor effect of anti-PD-1 was enhanced. When IL-18 was administered after anti-PD-1, little enhancement in the therapeutic effect of anti-PD-1 was observed (**Figure 1F**), indicating that IL-18 needs to be administered before anti-PD-1 to enhance the antitumor effect of anti-PD-1.

Our previous studies have shown that CD8^+^ T cells play an essential role in the therapeutic efficacy of combination therapy with IL-18 and ICB (anti-CTLA-4 and anti-PD-L1) (10). To investigate the relative contribution of NK cells and CD8^+^ T cells in the antitumor effect of anti-PD-1/IL-18, we generated β2-microglobulin (β2M)-deficient CT26 cells (CT26-Δβ2M) and compared their growth in mice with that of parental CT26 cells. As shown in **Figure 1G**, treatment with anti-PD-1/IL-18 slightly delayed the tumor growth of CT26-Δβ2M compared to parental CT26 cells. The experiment was terminated at day 30 because nearly all mice implanted with CT26-Δβ2M reached the humane endpoints due to tumor progression. These results indicate that cytotoxic T cells are essential for final tumor elimination in anti-PD-1/IL-18-treated mice. Consistent with this, CT26-bearing mice cured by anti-PD-1/IL-18 treatment established long-term protective immunity to the same CT26 tumors (**Figure 1H**). These results suggest that NK cells are a prerequisite for IL-18 to potentiate the antitumor effects of anti-PD-1, which ultimately requires cytotoxic T cells in different tumor models.

### 3.2 IL-18 activates NK cells in the tumor microenvironment under PD-1 blockade therapy and potentiates type 1 immune responses

**Figure 2:**
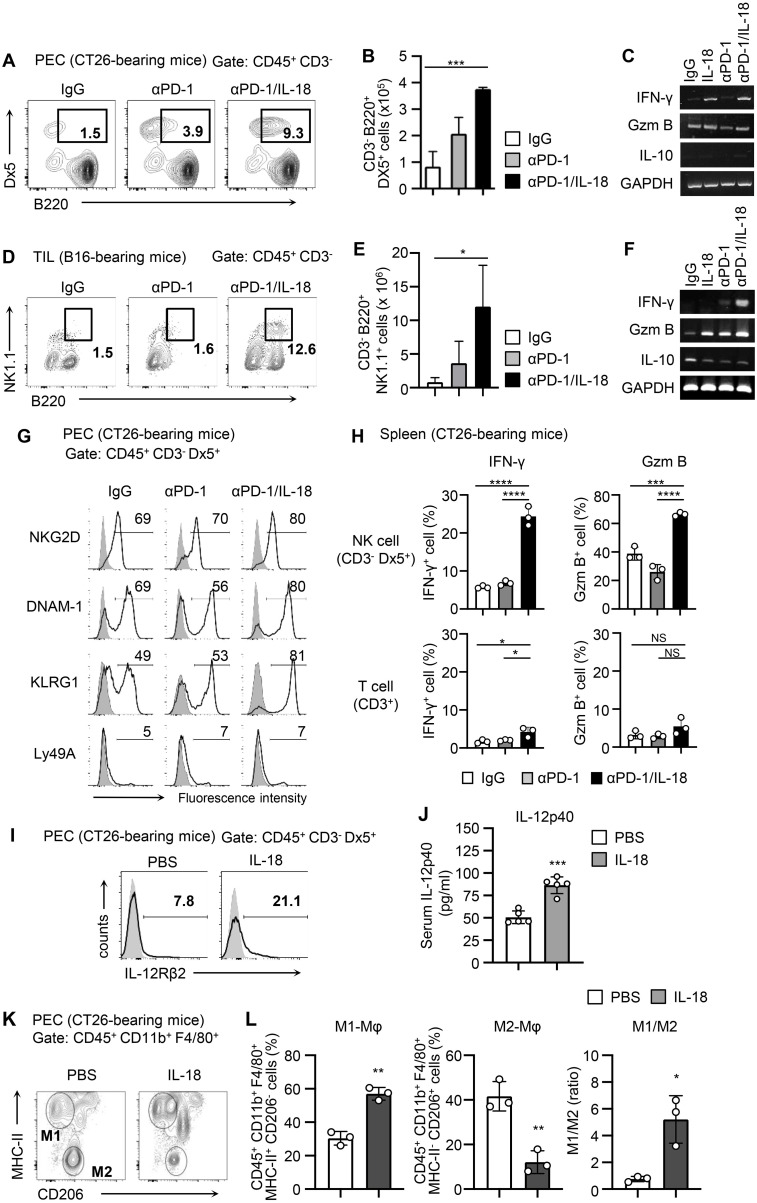
Anti-PD-1 mAb therapy with IL-18 activates NK cells and induces type 1 immune responses. **(A-F)** BALB/c mice were i.p. inoculated with CT26 cells (day 0) and i.p. treated with IL-18 or PBS as a control (day 3). C57BL/6 mice were s.c. inoculated with B16 cells and i.p. treated with αPD-1 and IL-18 as indicated when a tumor mass had formed. Accumulation of activated NK cells and expression of IFN-γ, granzyme B, and IL-10 at tumor sites (PECs on day 6 or TILs 3 days after administration) of tumor-bearing mice. Samples from CT26- **(A-C)** and B16- **(D-F)** bearing mice with or without anti-PD-1 and IL-18 i.p. administration were analyzed. Representative flow cytometric plots are shown in **(A-D)**, and the total numbers of activated B220^+^ NK cells were evaluated (B, E; N=3–4). The expression of IFN-γ, granzyme B, and IL-10 was analyzed by RT-PCR **(C, F)**. GAPDH was used as a control. **(G-L)** In the CT26-bearing BALB/c mice models, NK cells, T cells, and macrophages were analyzed. **(G)** On day 6, the activation of NK cells (CD45^+^ CD3^-^ Dx5^+^) in PECs were assessed by their expression of NKG2D, DNAM-1, Ly49D, Ly49H, KLRG1, siglec-E, and Ly49A. **(H)** IFN-γ and granzyme B expression on NK cells (CD3^-^ Dx5^+^) and T cells (CD3^+^) in the spleen were assessed by flow cytometry. **(I, J)** On day 6, the expression of IL-12 receptor β chains (IL-12Rβ) on NK cells (CD45^+^ CD3^-^ Dx5^+^) in PECs and serum IL-12 levels were assessed by flow cytometry and ELISA, respectively. **(K, L)** CT26-bearing mice were i.p. treated with IL-18 or PBS on days 3 and 6. On day 8, the phenotype of macrophages (CD45^+^ CD11b^+^ F4/80^+^) in PECs was assessed by their expression of MHC class II and CD206. Macrophages with M1 (MHC class II^+^/CD206^-^) or M2 (MHC class II^-^/CD206^+^) phenotypes were analyzed. **(K)** Representative flow cytograms and **(L)** proportion of macrophages with M1 or M2 phenotypes are shown along with M1/M2 ratios. Data were analyzed using ANOVA with multiple comparisons (Tukey *post hoc* analysis) **(B, E, H)** and an unpaired Student’s *t*-test **(J, L)**. Error bars indicate mean ± SD. ****, P < 0.0001; ***, P < 0.001; **, P < 0.01; *, P < 0.05.

To clarify the mechanism by which IL-18 enhances the antitumor effect of anti-PD-1 via NK cells, we analyzed the activation state of NK cells when anti-PD-1 was administered alone or in combination with IL-18 in the CT26 model. The number of activated B220^+^ NK cells among PECs of CT26-bearing mice was significantly increased in mice treated with anti-PD-1/IL-18 compared to those treated with control IgG or anti-PD-1 alone (**Figures 2A, B**). Upregulation of IFN-γ mRNA expression was also observed in PECs of CT26-bearing mice treated with anti-PD-1/IL-18 (**Figure 2C**). In the B16 model, similar increases in the number of activated B220^+^ NK cells and upregulation of IFN-γ were observed in tumor-infiltrating cells (TILs) after anti-PD-1/IL-18 administration (**Figures 2D–F**). Furthermore, treatment with anti-PD-1/IL-18 induced NKG2D, DNAM-1, and KLRG1 expression in NK cells in PECs (**Figure 2G**), and NK cells in the spleen showed significantly increased Granzyme B and IFN- γ expression (**Figure 2H**). In PECs of CT26-bearing mice, IL-18 treatment induced IL-12R expression in NK cells and increased IL-12 levels in the serum (**Figures 2I, J**). It also caused an increase in the population of M1-type macrophages and a decrease in M2-type macrophage population, and the M1/M2 ratio was significantly increased in PECs (**Figures 2K, L**). These observations suggest that IL-18 activates NK cells in the tumor microenvironment during PD-1 blockade therapy to enhance IFN-γ production and M1 macrophage polarization, thereby augmenting type 1 immune responses.

### 3.3 Sequential recruitment of NK cells and CD8^+^ T cells is necessary for PD-1 blockade therapy with IL-18

**Figure 3:**
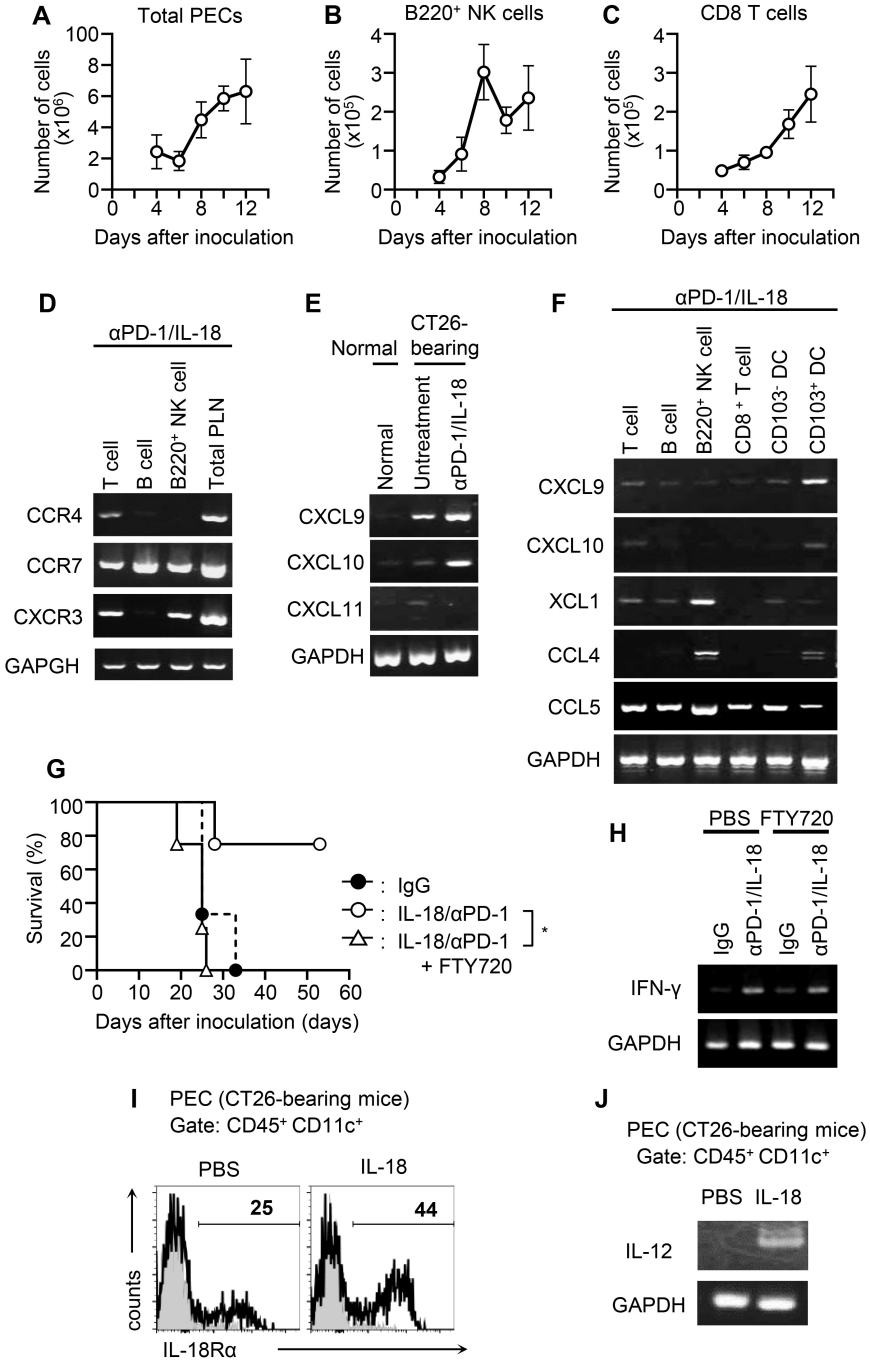
Sequential recruitment of activated NK cells and CD8^+^ T cells into tumor sites during anti-PD-1 mAb therapy with IL-18. **(A-C)** CT26 cells were i.p. inoculated (day 0) with or without anti-PD-1 + IL-18 i.p. administration as in **Figure 1A**, and the number of infiltrating cells in tumor sites at different time points was counted by flow cytometry. **(A)** Number of PECs. **(B, C)** Numbers of B220^+^ NK cells (B) and CD8^+^ T cells (C) in PECs. **(D-F)** Expression of chemokines and chemokine receptors in cells infiltrating tumor sites in mice treated with anti-PD-1 + IL-18 (day 6). **(D)** RT-PCR analysis of the expression of CCR4, CCR7, and CXCR3 in T cells, B cells, and B220^+^ NK cells in PECs. **(E, F)** Expression of chemokines (CXCL9, CXCL10, CXCL11, XCL1, CCL4, and CCL5) in cells infiltrating tumor sites. **(E)** Total PECs. **(F)** FACS-sorted T cells, B cells, B220^+^ NK cells, CD8^+^ T cells, CD103^-^ DCs, and CD103^+^ cDCs from PECs. **(G)** Effects of FTY720 on the survival of CT26-bearing mice i.p. treated with anti-PD-1 + IL-18. BALB/c mice were i.p. inoculated with CT26 (day 0) with or without anti-PD-1 + IL-18 i.p. administration on days 3, 7, 11, and 15. FTY720 was i.p. administered on days 2, 6, 10, and 14. **(H)** Effects of FTY720 on IFN-γ expression in PECs of CT26-bearing mice i.p. treated with anti-PD-1 + IL-18. PECs were collected on day 6 from mice treated as in (G). **(I, J)** BALB/c mice were i.p. inoculated with CT26 cells (day 0) and i.p. treated with IL-18 or PBS as a control (day 3). On day 6, DCs (CD45^+^ CD11c^+^) in PECs were assessed for the expression of IL-18Ra and IL-12 by flow cytometry and RT-PCR, respectively.

We then investigated the accumulation of immune cells at tumor sites in CT26-bearing mice treated with anti-PD-1/IL-18. We differentially counted B220^+^ NK cells and CD8^+^ T cells in PECs of CT-26 bearing mice and found that upon treatment with anti-PD-1/IL-18 on day 3, the number of activated B220^+^ NK cells peaked on day 6 followed by a gradual increase in numbers of CD8^+^ T cells (**Figures 3A–C**). Since chemokine signals function as cues for the coordinated recruitment of immune cells within and into tumor sites (26), we analyzed the expression of chemokines and chemokine receptors in PECs on day 6 of CT26-bearing mice treated with anti-PD-1/IL-18. We found that intraperitoneal (i.p.) B220^+^ NK cells from anti-PD-1/IL-18-treated mice expressed chemokine receptors such as CCR7 and CXCR3, whereas CCR4 expression was weakly detected in T cells in addition to CXCR3 and CCR7. In B cells, CCR7 expression was detected. (**Figure 3D**). Regarding chemokine expression in PECs, IFN-inducible chemokines including CXCL9 and CXCL10, which are involved in the mobilization of antitumor effector cells such as cytotoxic T cells and NK cells, were expressed (**Figure 3E**). Of these, CXCL9 expression was mainly detected in CD103^+^ DCs (**Figure 3F**). Regarding chemokines involved in cDC1 mobilization, XCL1, and CCL4 were expressed exclusively in B220^+^ NK cells. In contrast, CCL5 was detected in all cell types analyzed (**Figure 3F**).

The expression pattern of chemokines and chemokine receptors suggests the involvement of chemokine signaling in the sequential recruitment of NK cells and cytotoxic T cells. To better understand whether the observed migration of immune effector cells to the tumor site was linked with the antitumor effect of anti-PD-1/IL-18, we conducted experiments using FTY720, which inhibits lymphocyte egress from secondary lymphoid tissues. Administration of FTY720 largely inhibited the antitumor efficacy of anti-PD-1/IL-18 therapy (**Figure 3G**), whereas it did not significantly affect IFN-γ production at the tumor site (**Figure 3H**). These findings collectively suggest that in anti-PD-1 immunotherapy with IL-18, early accumulation of activated NK cells at the tumor site followed by subsequent mobilization of effector CD8^+^ T cells is necessary to exhibit antitumor effects of immunotherapy with anti-PD-1/IL-18.

### 3.4 IL-18-activated NK cells share premature phenotypes and functionally cooperate with DCs

Recent studies have identified distinct functional subsets of NK cells in the tumor microenvironment (27). Differentiating NK cells have been classified into four stages according to their expression of CD11b and CD27, but the relevant roles of these NK cell populations in antitumor immunity remain elusive (28). We thus further analyzed the properties of B220^+^ NK cells that appeared in tumor-bearing mice upon IL-18 stimulation. Analysis of NK cells in the spleens of mice treated with IL-18 revealed that most activated B220^+^ NK cells express CD27 and CD62L (**Supplementary Figures S2A–C**) and have a CD27^+^/CD11b^-^ phenotype, similar to premature NK cells (**Supplementary Figures S2D, E**).

Activated NK cells have been shown to crosstalk with DCs and play critical roles in initiating and regulating adaptive immunity against cancer (28). During the combined immunotherapy, IL-18 may stimulate both NK cells and DCs and facilitate functional crosstalk between NK cells and DCs. Indeed, a subset of DCs expressed IL-18R (**Figure 3I**), and injection of IL-18 upregulated IL-12 mRNA in CD11c^+^ DCs (**Figure 3J**). The presumed crosstalk is also supported by the fact that IL-18 in *in vitro* studies significantly upregulates IL-12 production in poly (I:C)-primed bone marrow-derived DCs (**Supplementary Figures S3A, B**). In NK cells, reciprocally, *in vitro* analyses in the presence of IL-15 showed that IL-18 upregulated IL-12R expression (**Supplementary Figure S3C**) and that IL-12 significantly induced XCL1 and CCL5 mRNA expression (**Supplementary Figure S3D**). Of note is that XCL1 mRNA expression induced in NK cells by IL-18 administration was inhibited by the neutralization of IL-12 by specific antibodies (**Supplementary Figure S3E**). These observations support the idea that IL-18 cooperatively stimulates NK cells and DCs and promotes NK–DC crosstalk involving IL-12 and XCL1 during PD-1 blockade therapy.

### 3.5 IL-18-activated NK cells recruit cDC1 into the tumor site during PD-1 blockade therapy and enhance the antitumor activity

**Figure 4:**
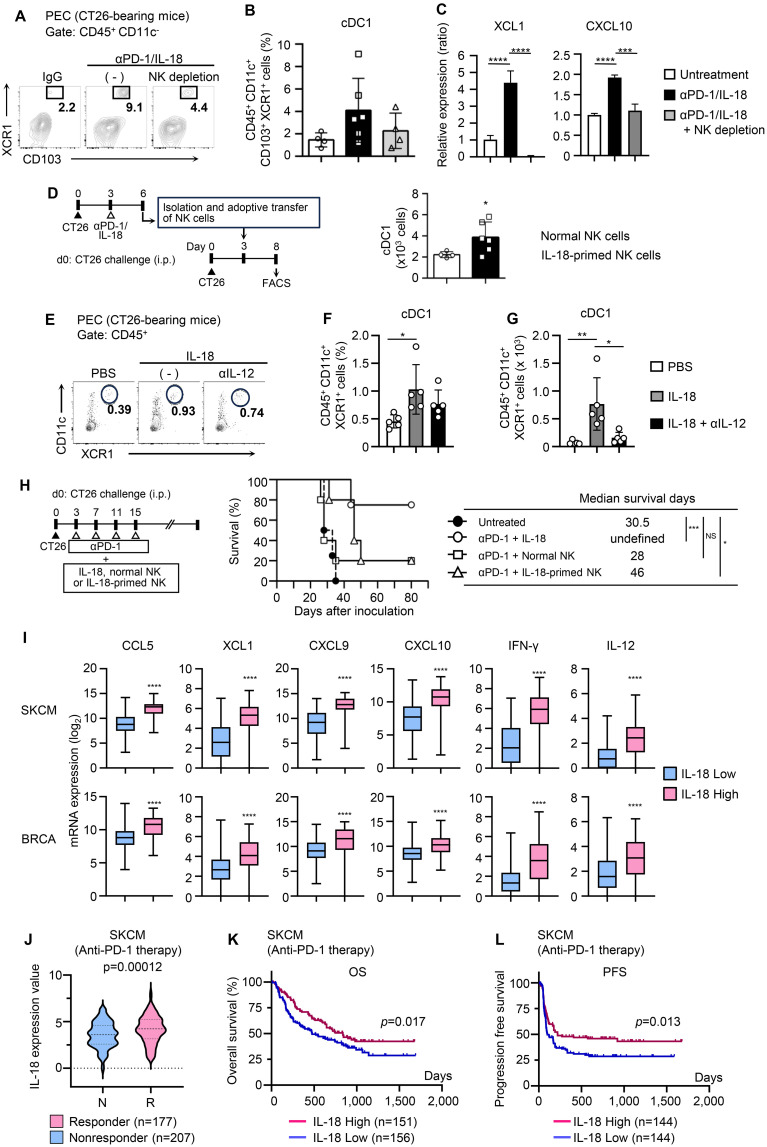
IL-18-primed NK cells mobilize cDC1s and enhance antitumor efficacy of PD-1 blocking immunotherapy. **(A, B)** Effects of NK cell depletion on the accumulation of cDC1 (CD103^+^ XCR1^+^ DCs) at tumor sites during anti-PD-1 + IL-18 therapy. CT26-bearing mice with or without αaGM1 i.p. injection were i.p. treated with anti-PD-1 + IL-18 as in **Figure 1A**. **(A)** PECs were collected on day 6, and cDC1 cells (CD45^+^ CD11c^+^ CD103^+^ XCR1^+^) were analyzed by flow cytometry. **(B)** Summarized FACS results (N=4–5) quantifying the percentages of cDC1 cells in PECs. **(C)** Effects of NK cell depletion on chemokine ligand expression in PECs. PECs were collected as in **(A)** and subjected to RT-PCR analysis for XCL1 and CXCL10 expression. **(D)** NK cells isolated from CT26-bearing mice with or without anti-PD-1 + IL-18 i.p. administration were i.p. transferred into different CT26-bearing mice and analyzed for cDC1 accumulation in PECs. (Left) Experimental design. (Right) Numbers of accumulated cDC1s in PECs. Summarized results (N=4–6) are shown. **(E-G)** Effects of anti-IL-12 antibody on cDC1 accumulation induced by IL-18. BALB/c mice were i.p. inoculated with CT26 cells (day 0) and i.p. treated with IL-18 (day 3). The accumulation of cDC1s in PECs was assessed by flow cytometry (day 6). To inhibit IL-12, neutralizing anti-IL-12 antibody was i.p. injected on day 2. PBS was used as a control. **(E)** Representative flow cytogram. The proportions **(F)** and the cell number **(G)** of cDC1s (CD45^+^ CD11c^+^ CD103^+^ XCR1^+^) in PECs are shown**. (H)** Comparison of antitumor effects of anti-PD-1 combined with either IL-18, unprimed NK cells, or IL-18-primed NK cells on survival of CT26-bearing mice. (Right) Experimental design. (Middle) Survival time analysis for CT26-bearing mice i.p. treated with anti-PD-1 in combination with IL-18 (N=5), control NK cells (N=5), or IL-18-primed NK cells (N=5). The median survival time (days) is shown (right). **(I)** Skin cutaneous melanoma (SKCM) and breast invasive carcinoma (BRCA) datasets were retrieved from cBioPortal and analyzed with Prism 6.0. Expression analyses (*CCL5, XCL1, CXCL9, CXCL10 IFNγ, and IL12*) are shown. **(J-L)** IL-18 expression and disease outcomes. Aggregated datasets of patients with SKCM who had received anti-PD-1 therapy were obtained from Cancer-Immu and processed with Prism 6.0 **(J)**. IL-18 expression in responders and non-responders. Kaplan-Meier analysis of overall survival **(K)** and progression-free survival **(L)** in patients with high or low IL-18 expression. Data were analyzed using ANOVA with multiple comparisons (Tukey *post hoc* analysis; **B, C, F, G**), unpaired Student’s *t*-test (**D, I, J**), and log-rank test (**H, K, L**). Error bars indicate mean ± SD. ****, P < 0.0001; ***, P < 0.001; **, P < 0.01; *, P < 0.05.

We then sought to elucidate the mechanism by which IL-18-activated NK cells enhance the therapeutic effect of anti-PD-1. Because NK cells in tumor-bearing mice treated with anti-PD-1/IL-18 expressed multiple chemokines that contribute to cDC1 mobilization such as XCL1 and CCL4, we investigated whether IL-18-activated NK cells were directly involved in cDC1 recruitment into tumor sites. As shown in **Figures 4A, B**, the depletion of NK cells strongly reduced the accumulation of XCR1^+^ CD103^+^ cDC1s in the peritoneal cavity of CT26-bearing mice. Consistent with this, NK cell depletion reduced the production of chemokines, including XCL1 and CXCL10, in PECs (**Figure 4C**).

We then tested whether IL-18-primed NK cells directly contribute to cDC1 recruitment into tumor sites and enhance the efficacy of anti-PD-1. To this end, we prepared untreated or *in vivo* primed NK cells with IL-18, injected them into CT26-bearing mice, and compared their effects on cDC1 recruitment. As shown in **Figure 4D**, i.p. injection of IL-18-primed NK cells significantly increased the number of cDC1 in PECs of CT26-bearing mice compared to the injection of unprimed NK cells. In addition, results obtained with the neutralizing antibody to IL-12 showed that the IL-18-induced recruitment of cDC1 was IL-12-dependent (**Figures 4E–G**). Finally, we examined whether IL-18-activated NK cells potentiate the antitumor effects of PD-1 blockade therapy. Albeit less pronounced, injection of IL-18-primed NK cells enhanced the therapeutic efficacy of anti-PD-1 in CT26-bearing mice, and the mean survival time was prolonged from 28 days (anti-PD-1 + unprimed NK cells) to 46 days (anti-PD-1 + IL-18-primed NK cells) (**Figure 4H**). Together, these results showed that IL-18-primed NK cells play critical roles in cDC1 recruitment into tumor sites and enhance the antitumor immune response induced by PD-1 blockade. Analyses using publicly available clinical data of patients with skin cutaneous melanoma (SKCM) and breast cancer (BRCA) showed that the intensity of IL-18 expression correlated with the expression of inflamed tumor-related genes, involving NK–DC crosstalk (CCL5 and XCL1), effector T-cell recruitment (CXCL9 and CXCL10), and type 1 immune response (IL-12 and IFN-γ), as well as the levels of infiltration of activated NK cells and CD8^+^ T cells into the cancer tissues (29), supporting this notion (**Figure 4I**, **Supplementary Figures S3F, G**).

The results of this study altogether suggest that in the combined treatment of IL-18 and anti-PD-1, IL-18 acts on NK cells to promote cDC1 mobilization to tumor sites to initiate type 1 immune responses and enhance the antitumor effect of PD-1 blockade. In this process, IL-18 may also act on DCs to promote IL-12 production (**Figure 3J**), facilitating the formation of a feed-forward loop by NK cells and DCs that promotes the production of DC-attracting chemokines such as XCL1 and CCL5 by IL-18-primed NK cells (**Figure 4C**, **Supplementary Figure S3D**). Antibody blocking studies have shown that IL-18-mediated mobilization of cDC1 to the tumor site is IL-12-dependent, supporting this possibility. A recent study using a recombinant adenovirus platform also reported that intratumor administration of IL-12 augments the production of the DC-attracting chemokine CCL5 by tissue-resident NK cells and enhances antitumor immunity (30). In the clinical phenotypes of SKMC patients treated with anti-PD-1 (19), IL-18 expression was higher in the responder group than in the non-responder group (**Figure 4J**), and the high IL-18 expression group showed survival advantages (**Figures 4K, L**), suggesting that IL-18 acts as a factor that enhances the antitumor effect of anti-PD-1 antibodies in certain clinical settings. The recently developed genetically engineered variant IL-18 without binding activity to IL-18BP, which is an endogenous IL-18 inhibitor, has been shown to potentiate the action of IL-18 and enhance the therapeutic effect of checkpoint inhibitors (12).

However, this study has several limitations. First, the mechanism of mobilization of cDC1s into tumor tissue by IL-18-primed NK cells is not clear; IL-18-primed NK cells express several chemokines, which may be involved alone or in combination in the mobilization of DCs into tumor tissue. Second, IL-18-primed NK cells can only partially account for the enhanced action of immune checkpoint inhibitors by IL-18; IL-18 may act on a certain T cell population(s) as well as DCs in addition to NK cells, and these cells may all work in concert to enhance the anti-tumor immune response by IL-18. These issues should be clarified in future studies.

This study’s results highlight the capability of IL-18 in enhancing NK-DC crosstalk during PD-1 blocking therapy, and the importance of the priming step of NK cells in potentiating antitumor effects of immune checkpoint inhibitors. Our findings provide an additional strategy for potentiating the antitumor effects of immune checkpoint inhibitors, especially when tumors have inadequate or resistant immune responses.

## Data Availability

The original contributions presented in the study are included in the article/**Supplementary Material**, further inquiries can be directed to the corresponding author.
