# Supplementary material for: IL-18-primed NK cells recruit dendritic cells and potentiate tumor therapy mediated by PD-1 blockade
Source: Front Oncol. 2025 Mar 17;15:1533808. doi: 10.3389/fonc.2025.1533808 (PMC11955453; doi:10.3389/fonc.2025.1533808)
Supplement: Supplementary file 1 [file DataSheet1.pdf]

Supplemental Figure 1

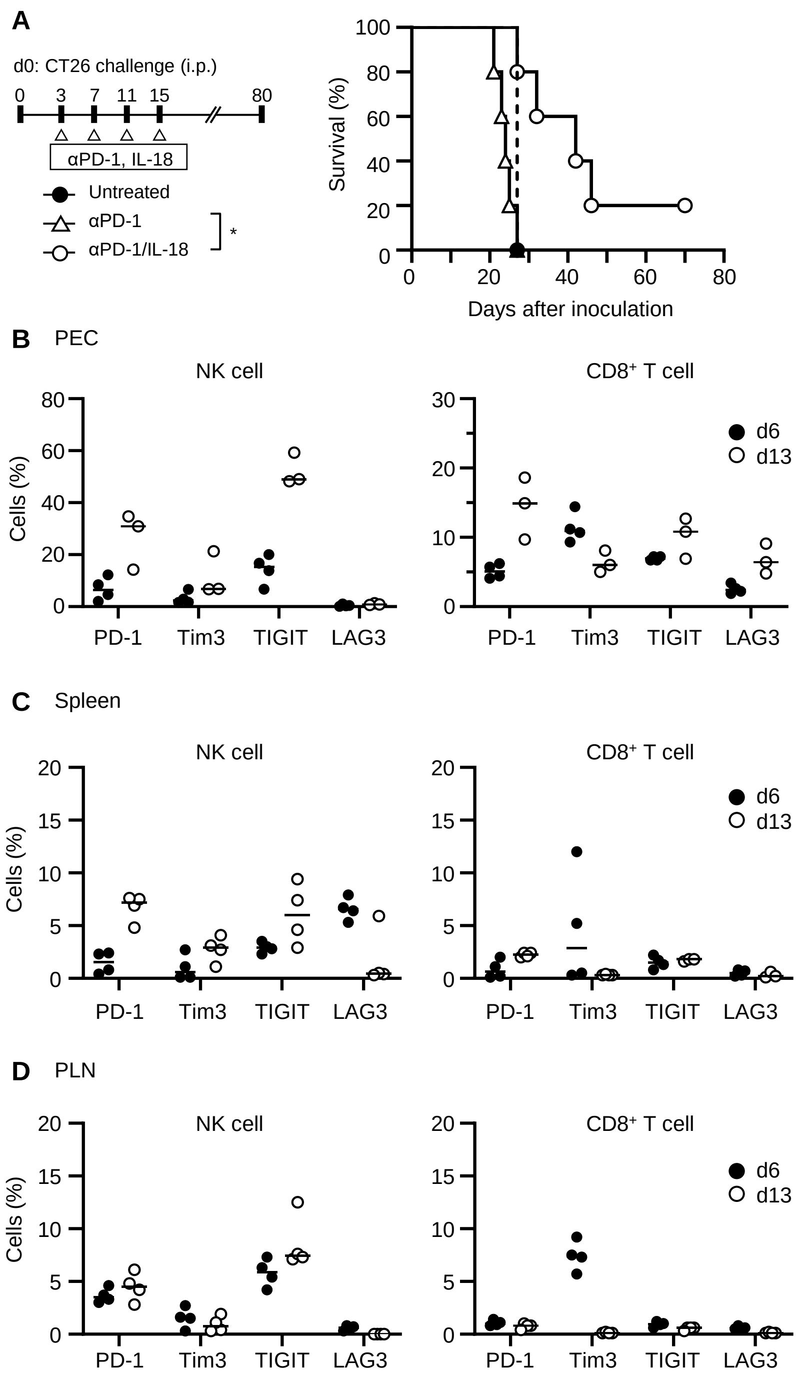

Figure S1. Expression of checkpoint molecules on NK cells and T cells in tumor sites and secondary lymphoid tissues following treatment with anti-PD-1 + IL-18. (A) Effects of IL-18 on the therapeutic efficacy of anti-PD-1. BALB/c mice were i.p. inoculated with CT26 cells (day 0) with or without anti-PD-1 i.p. administration alone or in combination with IL-18 on days 3, 7, 11, and 15. The survival of these mice was analyzed. **(B-D)** BALB/c mice were i.p. inoculated with CT26 cells (day 0). On day 6 and day 13, the expression of PD-1, Tim3, TIGIT, and LAG3 on NK cells and $CD8^+$ T cells in PECs (B), the spleen (C), and peripheral lymph nodes (D, PLNs) were analyzed using fluorescently conjugated antibodies (anti-CD3 (145-2C11), anti-CD8 (53-6.7), anti-CD49b (Dx5), anti-PD-1 (29F.1A12), anti-Tim3 (B8.2C12), anti-TIGIT (1G9), and anti-LAG3 (C9B7W) all BioLegend) by flow cytometry. Dead cells were stained by DAPI and excluded from the analysis. Data were analyzed using the log-rank test. *, $P < 0.05$.

Supplemental Figure 2

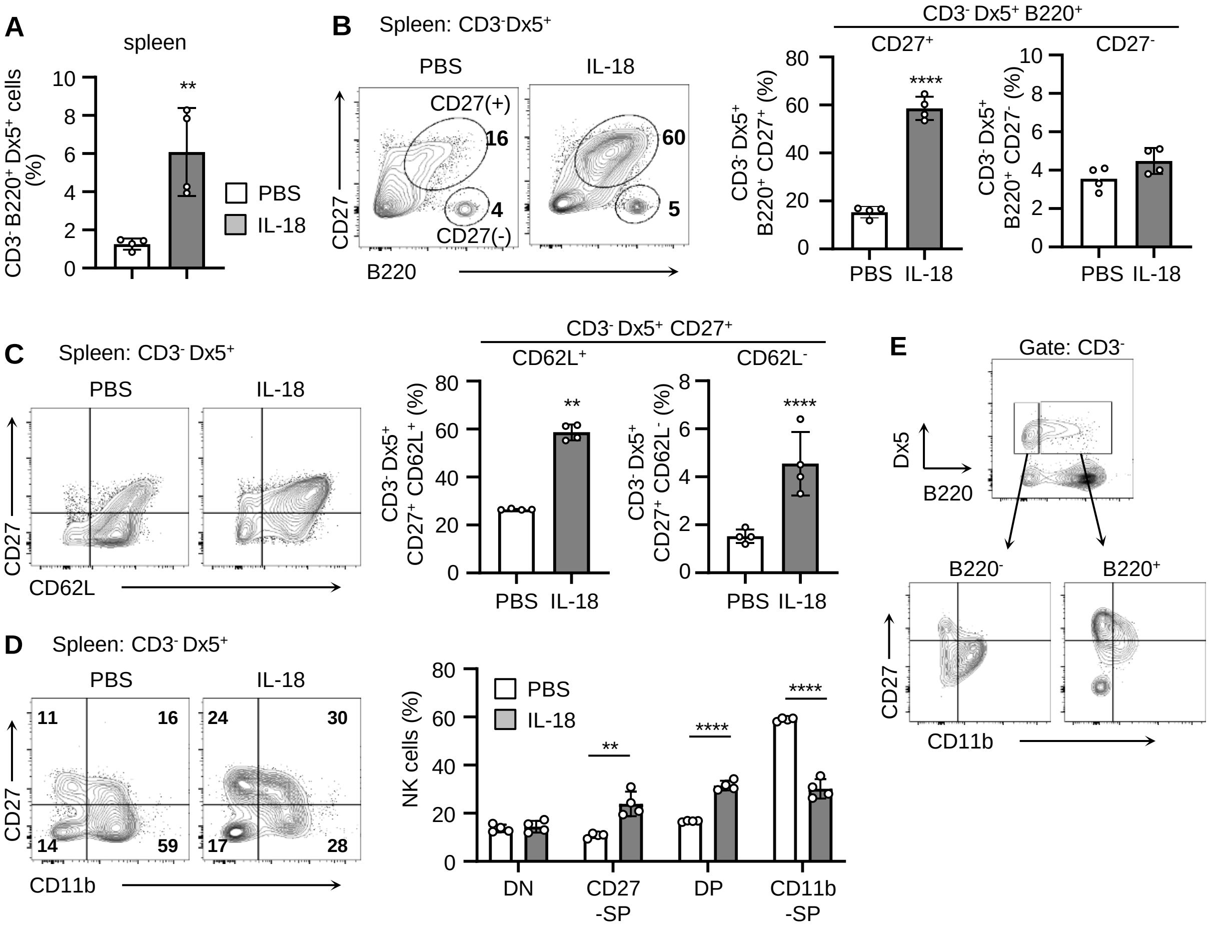

Figure S2. IL-18-primed NK cells. Naïve Balb/c mice were i.p. injected with IL-18 (1 μg/mouse) or PBS. After 3 days, splenic NK cells were analyzed using flow cytometry. Activated NK cells ($CD3^-$ $B220^+$ $Dx5^+$) were expanded by IL-18 i.p. administration **(A)**. The expression of CD27 **(B)** and CD62L **(C)** on IL-18-activated NK cells was assessed by flow cytometry. **(D)** The phenotype of the activated NK population in the spleen of mice i.p. treated with IL-18. The differentiation status of NK cells was assessed by CD27 and CD11b expression. The representative cytogram (left) and the frequency of each subset of NK cells (right, N=3–4) are shown. **(E)** $B220^-$ or $B220^+$ NK cells were assessed for their expression of CD27 and CD11b. Data were analyzed using the unpaired Student's *t*-test. Error bars indicate mean ± SD. ****, P < 0.0001; **, P < 0.01.

Supplemental Figure 3

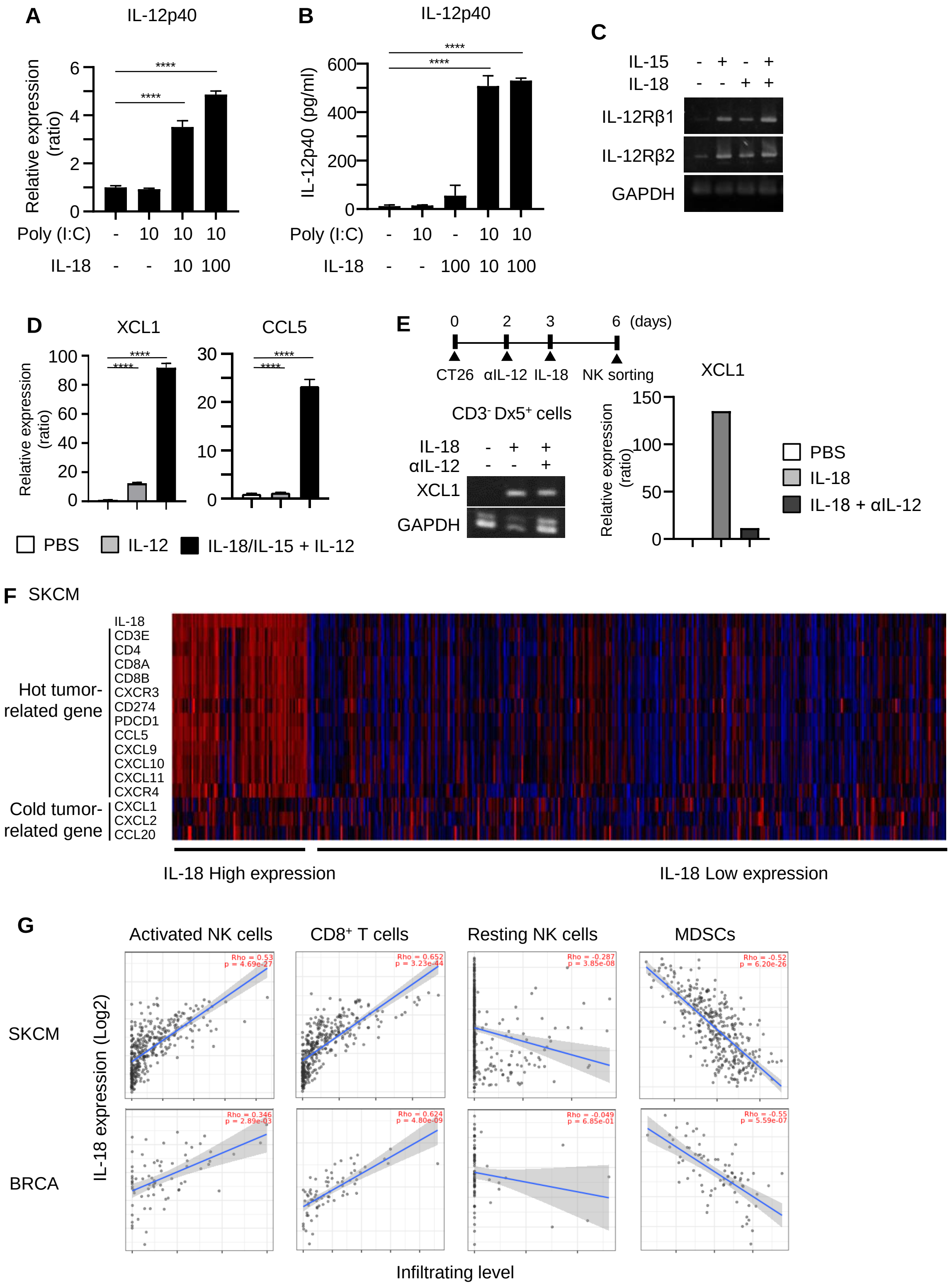

Figure S3. IL-18 activates the NK-DC axis and promotes type 1 immune responses. (A, B) Enhancement of poly(I: C)-induced IL-12 expression by IL-18. Bone marrow-derived DCs (BM-DCs) were stimulated with poly(I: C) (10 μg/mL) with or without IL-18 (10 or 100 ng/mL) for 18 h, and then IL-12p40 mRNA expression (A) and IL-12p40 protein in the culture supernatant (B) were quantified by real-time PCR and ELISA, respectively. **(C)** NK cells were isolated from naïve BALB/c mice and cultured in the presence of IL-15 (10 ng/mL) with or without IL-18 (100 ng/mL). IL-12 receptor (β1 and β2) expression on activated NK cells was assessed by RT-PCR. (**D**) Mouse NK cells were preactivated with or without IL-18/IL-15 for 48 h and then further stimulated with IL-12 for 18 h. The chemokine (*Xcl1* and *Ccl5*) induction by IL-12 in naïve or IL-18/IL-15-preactivated NK cells was assessed by real-time PCR. (**E**) BALB/c mice were i.p. inoculated with CT26 cells (day 0) and i.p. treated with anti-IL-12 on day 2 and IL-18 (1 μg/mouse) on day 3. On day 6, NK cells ($CD45^+$ $CD3^-$ $Dx5^+$) in PECs were sorted using FACSAriaIIIu. *Xcl1* mRNA expression in NK cells was analyzed by RT-PCR. The intensity of the bands was quantified by densitometry using Image J and normalized to *Gapdh* expression. (**F**) Heatmap analysis of skin cutaneous melanoma (SKCM) datasets retrieved from cBioPortal. (**G**) Correlation analyses of the levels of infiltrating immune cells (activated NK cells, $CD8^+$ T cells, resting NK cells, and myeloid-derived suppressor cells [MDSCs]) and IL-18 expression in SKCM and BRCA datasets were performed using TIMER2.0 (http://timer.cistrome.org/). Data were analyzed using ANOVA with multiple comparisons (Tukey post hoc analysis) (A, B, D). Error bars indicate mean ± SD. ****, $P < 0.0001$.
